# Mapping immunogenic epitopes of an adhesin-like protein from *Methanobrevibacter ruminantium* M1 and comparison of empirical data with in silico prediction methods

**DOI:** 10.1038/s41598-022-14545-8

**Published:** 2022-06-21

**Authors:** Sofia Khanum, Vincenzo Carbone, Sandeep K. Gupta, Juliana Yeung, Dairu Shu, Tania Wilson, Natalie A. Parlane, Eric Altermann, Silvia M. Estein, Peter H. Janssen, D. Neil Wedlock, Axel Heiser

**Affiliations:** 1 AgResearch, Palmerston North, New Zealand; 2 Riddet Institute, Massey University, Palmerston North, New Zealand; 3 Centro de Investigación Veterinaria de Tandil (CIVETAN), UNCPBA-CONICET-CICPBA, Facultad de Ciencias Veterinarias, Campus Universitario, 7000 Tandil, Argentina

**Keywords:** Immunology, Biochemistry, Peptides, Proteins, Computational biology and bioinformatics, Protein structure predictions

## Abstract

In silico prediction of epitopes is a potentially time-saving alternative to experimental epitope identification but is often subject to misidentification of epitopes and may not be useful for proteins from archaeal microorganisms. In this study, we mapped B- and T-cell epitopes of a model antigen from the methanogen *Methanobrevibacter ruminantium* M1, the Big_1 domain (AdLP-D1, amino acids 19–198) of an adhesin-like protein. A series of 17 overlapping 20-mer peptides was selected to cover the Big_1 domain. Peptide-specific antibodies were produced in mice and measured by ELISA, while an in vitro splenocyte re-stimulation assay determined specific T-cell responses. Overall, five peptides of the 17 peptides were shown to be major immunogenic epitopes of AdLP-D1. These immunogenic regions were examined for their localization in a homology-based model of AdLP-D1. Validated epitopes were found in the outside region of the protein, with loop like secondary structures reflecting their flexibility. The empirical data were compared with epitope predictions made by programmes based on a range of algorithms. In general, the epitopes identified by in silico predictions were not comparable to those determined empirically.

## Introduction

Methane accounts for almost a third of New Zealand’s anthropogenic greenhouse gas emissions, with 80% of these emissions coming from enteric fermentation of ruminant livestock^1^. An anti-methanogen vaccine could be a good option to target methane-producing methanogens in the rumen, thereby reducing methane emissions. A vaccine could induce neutralizing antibodies in saliva^2^ which bind to surface antigens of methanogens in the rumen and inhibit their growth and thereby reduce methane production^3^. While IgA is the major class of antibody in saliva, our trials vaccinating sheep and cattle with methanogen antigens have generated predominately antigen-specific IgG in saliva^4,5^. Thus, in this study, we focused on mapping IgG1 and IgG2a epitopes. A crucial step for the development of an effective vaccine is the discovery of suitable antigens, which include surface located proteins with immunological epitopes accessible for antibody binding.

The use of short immunogenic peptides as vaccine antigens instead of entire proteins may produce a more targeted immune response. In recent years, epitope mapping has emerged as a tool for screening and selection of antigenic peptide to design effective peptide-based vaccines^6,7^. Active immune responses induced by antigenic peptides are mediated by B and T lymphocytes. These lymphocytes recognize a specific region on their cognate antigens known as epitopes, through their specialized receptors. B-cells recognize protein antigens in their native structure based on their linear or conformational epitope. Several experimental techniques are available to identify B-cell epitopes, such as X-ray crystallography, phage and yeast display methods, partial proteolysis, mass spectrometry, mutagenesis, and peptide screening^8^. On the other hand, T-cells only recognize linear peptide fragments of antigens presented by various MHC molecules on antigen-presenting cells (APC). T-cell epitopes have been identified using experimental techniques such as intracytoplasmic cytokine staining, enzyme-linked immune absorbent spot (ELISpot), in vitro stimulation, and proliferation of immune cells^9^. However, such experimental techniques of B- and T-cell epitopes identification have limitations due to their ability to differentiate between conformational and linear epitopes and requirement of relatively large amounts of purified protein/antibody complexes together with their structures. Therefore, caution must be taken in analyzing the results acquired by different methods. Prediction of immunogenic epitopes could be enhanced and confirmed by combining two or more techniques.

In silico prediction of epitopes is a potentially time-saving alternative to experimental epitope identification but can result in the misidentification of epitopes^9–11^. Several immunoinformatics tools have been developed for the prediction of antigenic B- and T-cell epitopes^9^. Current methods for predicting linear T-cell epitopes screen for the shortest peptide sequence recognized by the MHC class I and MHC class II binding sites. BALB/c mice used in this study for epitope mapping express three MHC class I (H2-Kd, H2-Ld and H2-Dd) and two MHC class II (I-Ad and I-Ed) molecules. Epitope specificity can also be modified by a proteolytic process by which protein antigens are cleaved into peptide fragments within APCs, depending on the class of APC as well as its activation status^12^. Different algorithms are available to predict linear epitope sequences for T-cell epitopes, such as Rankpep^13^, SYFPEITHI^14^ and IEDB^15^. Rankpep and SYFPEITHI are based on binding motifs of peptides to MHC class I or II alleles and their proteasome cleavage specificities. IEDB is based on combined methods of quantitative affinity matrices (QAMs) and artificial neural networks (ANNs) and corresponds to predicted binding affinities of peptide binding to MHC I and II molecules^16^.

Linear B-cell epitope prediction is similar to T-cell epitope prediction and is primarily based on amino acid properties such as hydrophilicity, flexibility, charge, exposed surface area and secondary structure. To enhance the performance of linear B-cell epitope predictions, machine learning-based algorithms have been developed to distinguish experimental B-cell epitopes from non-B cell epitopes. Relevant examples of linear B-cell epitope prediction methods are BepiPred^17^, ABCpred^18,19^, BCpreds^19^, and SVMtrip^20^ using different datasets, training features and algorithms. BepiPred B-cell epitope prediction is based on a random forest algorithm trained on 3D-structures of antigen–antibody complexes, while BCpreds and SVMtrip are based on support vector machines (SVM) and ABCpred methods consist of artificial neural networks (ANNs)^9^.

Earlier attempts at computational prediction gave high false positive rates resulting in underuse of these methods^11,21^. However, this approach remains a potentially valuable time-saving alternative to experimental identification. Currently, the reliability of computational epitope predictions for archaeal proteins is unknown. Therefore, the present study aimed to map empirically the B- and T-cell epitopes of the Big_1 domain (amino acids 19–198) of adhesin like protein Mru_1499 of the methanogen *Methanobrevibacter ruminantium* M1. Big_1 forms part of the extracellular domain of the protein involved in protein-to-protein interactions^22^. A series of 17 overlapping 20-mer peptides was selected to cover the Big_1 domain and used to evaluate immune responses in mice. Peptide-specific antibodies were measured by ELISA and an in vitro splenocyte re-stimulation assay used for determining specific T-cell responses. The results were compared with in silico predictions of immunological epitopes.

## Results

### Production of AdLP-D1 and 20-mer peptides

**Figure 1:**
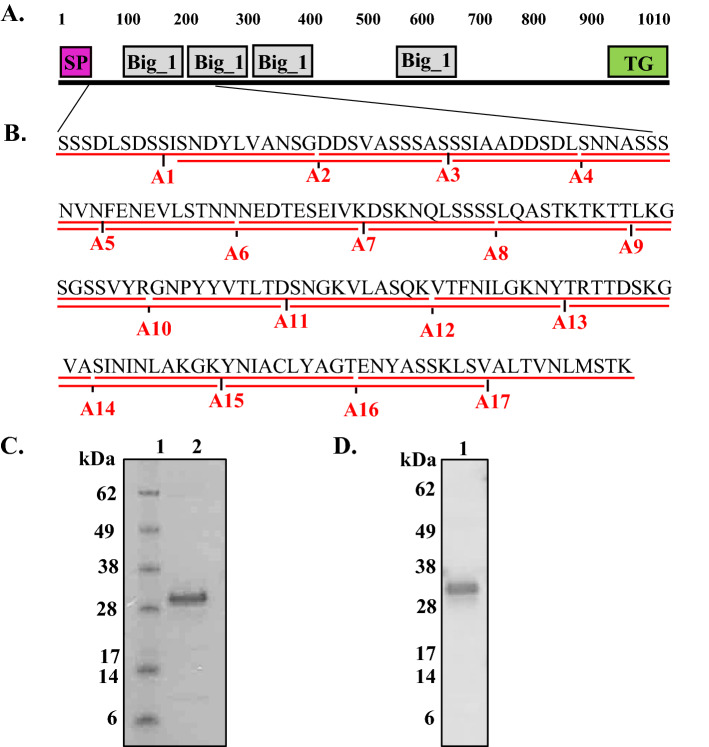
Characteristics and production of protein and peptides. (**A**) Domain organization of Mru_1499 of *Methanobrevibacter ruminantium* M1. Predicted domain architecture using SMART database and InterPro^22–24^. Signal peptide (SP) encoded by amino acids 1–18, four bacterial immunoglobulin class 1 (Big_1) domains and transglutaminase (TG) domain are represented as pink, grey and green rectangles, respectively. Numbers represent the amino acid residues in the protein sequence. (**B**) Schematic presentation of peptide design of AdLP-D1. Peptides of twenty amino acids long with 10 amino acids overlap are shown as red lines under the residues with the peptide designations in red. (**C**) Purified AdLP-D1 analyzed using SDS-PAGE, stained with Coomassie blue. Lane 1, SeeBlue Plus2 Pre-Stained Standard (Invitrogen, Carlsbad, CA, USA); lane 2, purified fraction of AdLP-D1. The complete gel is shown in Supplementary Fig. 1. (**D**) Western blot analysis of AdLP-D1 purified fraction using anti-hexahistidine-tag antibodies. The complete gel is shown in Supplementary Fig. 2.

Using SMART^23^ and InterPro^24^, the adhesin-like protein Mru_1499 of *Methanobrevibacter ruminantium* M1 has been reported in a previous study^22^ to contain three tandem Big_1 domains at residues 102–197, 195–283, and 286–390 and a fourth Big_1 domain, at residues 577–665 in the middle of the protein, and a transglutaminase-like domain at residues 878–981 (Fig. 1A). In the present study, we selected a part of this protein covering residues 19 to 198 of Mru_1499, which includes the first Big_1 domain, and is referred to as AdLP-D1 (Fig. 1B). A total of 17 overlapping peptides, each of twenty amino acids with 10 amino acids overlap of the next adjacent peptides, were designed to cover the AdLP-D1 sequence (Fig. 1B). Hexahistidine-tagged recombinant AdLP-D1 was produced in *Escherichia coli* and purified by affinity chromatography. A dominant band with an apparent size of 28 kDa was observed by SDS-PAGE analysis (Fig. 1C), which was larger than the expected molecular weight of the histidine-tagged AdLP-D1 (20 kDa). The 28 kDa protein band was confirmed as AdLP-D1 by Western blotting using anti-His tag antibodies (Fig. 1D) and mass spectrometry with more than 80% coverage.

### B-cell epitopes mapped by peptide-specific antibody response

**Figure 2:**
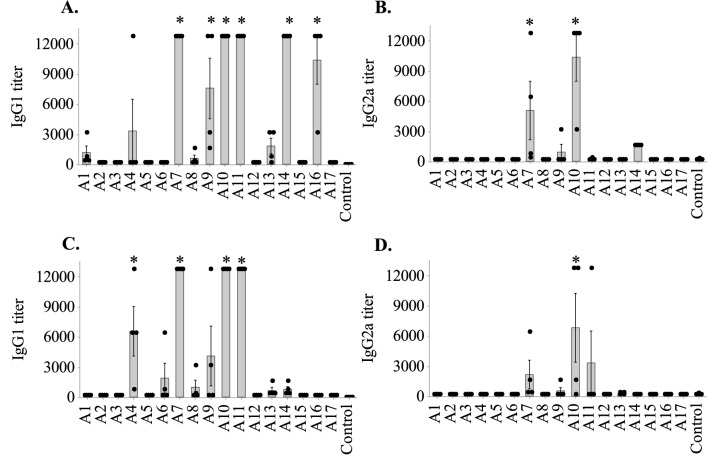
IgG1/IgG2a titers in mice vaccinated with the peptides and the recombinant AdLP-D1 protein. Mouse sera were tested for the presence of peptide-specific antibodies and their ability to bind to the recombinant AdLP-D1 protein. Individual data points are represented as black dots, while bars represent the geometric mean (± SE) of sera from four mice vaccinated with each peptide (A1 to A17). (**A**,**B**) antibody titers against the respective peptides used for the vaccination. (**C**,**D**) Antibody titers against the recombinant protein. Significance differences to the control (adjuvant only) group are shown as *P < 0.05.

To map the B-cell epitopes of AdLP-D1, mice were immunized with KLH-conjugated peptides. Sera were tested for presence of IgG1 and IgG2a antibodies against the corresponding vaccinated peptides to measure each peptide’s ability to generate a peptide-specific-antibody response. Significantly high IgG1 antibody responses were observed for peptides A7, A9, A10, A11, A14 and A16 in comparison to control mice that received only adjuvant, when their sera were tested against their corresponding peptides coated on ELISA plates (Fig. 2A). In contrast, only A7 and A10 peptides induced IgG2a antibody responses in mice (Fig. 2B).

To investigate the regions of the AdLP-D1 protein that were recognized by mouse sera generated against the peptides, ELISA plates were coated with recombinant AdLP-D1 protein and tested for IgG1 and IgG2a antibody responses. Significant IgG1 binding was observed to the recombinant protein with the sera produced against peptides A4, A7, A10 and A11, while sera from the control mice showed no detectable binding (Fig. 2C). IgG2a binding to the recombinant protein was observed with the serum raised against the A10 peptide (Fig. 2D). These results indicated that AdLP-D1 had two strongly immunogenic regions, corresponding to peptide A7 (residues 79–98) and peptides A10 and A11 (residues 109–138). A region of the protein, corresponding to peptide A4 (residues 49–68) was less immunoreactive. Vaccination with the AdLP-D1 protein itself resulted in high titers of IgG1 and IgG2a (Supplementary Fig. 3). These responses were higher than those measured against individual peptides, which is expected as the protein has multiple linear epitopes and likely contains conformational epitopes.

### T-cell epitopes mapped by peptide-specific cytokine response

We also tested the 17 peptides for their ability to induce cell-mediated immunity. Since the main focus of this study was mapping the immunogenic epitopes, we decided to include all T cells for which IFNγ appeared to be the suitable read-out. Therefore, immune responses were assessed by measuring the release of antigen-specific cytokines IFNγ and IL-17A from splenocytes of the vaccinated mice.

**Figure 3:**
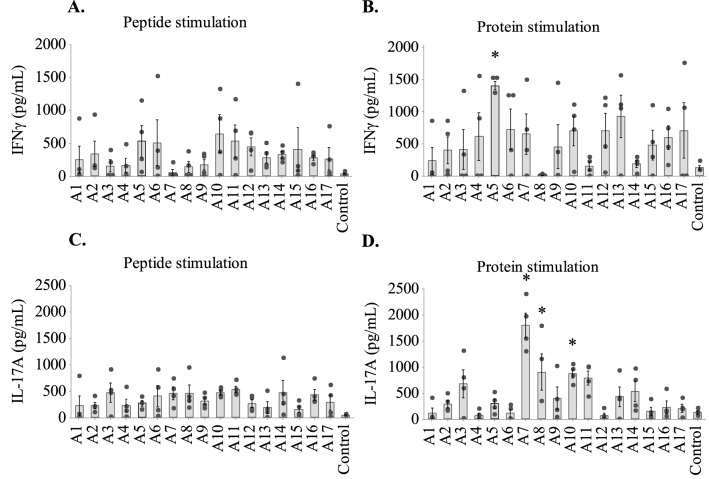
IFNγ and IL-17A levels in stimulated mouse splenocyte culture in vitro. IFNγ levels in the supernatants of (**A**) peptide-stimulated and (**B**) protein-stimulated splenocyte cultures. IL-17A concentration in supernatants of (**C**) peptide-stimulated and (**D**) protein-stimulated splenocyte cultures. Black dots represent individual data points of the IFNγ and IL-17A levels of individual mice. Bars represent the geometric mean (± SE) of culture supernatant from four mice vaccinated with each peptide. Significance differences to control (adjuvant only) group are shown as *P < 0.05.

Splenocytes were isolated from the vaccinated mice two weeks after the last vaccination and re-stimulated in vitro with the corresponding peptide or the recombinant protein^25^. Splenocytes of mice vaccinated with peptides A5, A6, A10, A11 and A15 induced IFNγ production upon re-stimulation with their corresponding vaccinated peptides (Fig. 3A). When splenocytes of the same batches were re-stimulated with recombinant AdLP-D1 separately, with the exception of peptide A5, there was non-significant induction of IFNγ in comparison to the control group (Fig. 3B). However, splenocytes from three out of four mice vaccinated with peptide A13 gave strong IFNγ responses to AdLP-D1 stimulation (IFNγ levels greater than 1000 pg/mL, *P* = 0.059), suggesting peptide A13 can be considered a T-cell epitope of AdLP-D1 (Fig. 3B). Peptides A5 and A13 corresponded to residues 59–78 and 139–158 respectively, indicating binding affinities of these regions of AdLP-D1 toward MHC class II molecules. Compared to the IFNγ responses, levels of IL-17A induction were more even among all the splenocyte samples re-stimulated with their corresponding peptides. Upon re-stimulation with AdLP-D1, splenocytes from mice vaccinated with the A7 peptide generated significantly high levels of IL-17A compared to the control, while IL-17A levels induced by A8, A10 and A11 peptides were also elevated (Fig. 3C,D). Collectively, the results suggested that regions covered by peptides A5, A7, A8, A10 and A13 are potential T-cell epitopes of the AdLP-D1 protein with binding affinity toward MHC class I and II molecules.

### Physiochemical properties of peptides

We analyzed the physiochemical properties of the peptides used to generate B- and T-cell responses using a range of predictive software to understand their shared features (Table 1) and facilitate the peptide selection process for vaccination. The ProtParam^26^ server was used to analyze physiochemical properties of peptides including theoretical isoelectric point (pI), charged amino acids composition, stability, aliphatic index and hydropathy (GRAVY) value. The value of pI ranging 3.42–4.18 exhibited by N-terminal peptides of AdLP-D1 (A1–A7) revealed that these peptides are acidic in nature, while peptides covering the C-terminal end of AdLP-D1 (A8–A17) are basic, having pI > 9. Instability indices of these peptides were variable, ranging from mostly stable peptides (A10 and A11) with instability indices of − 9.59 and 9.00, respectively, to highly unstable peptides (A15 and A16) with instability indices of 84.14 and 97.52, respectively. Peptides covering the N and C-terminal ends of AdLP-D1 were mostly unstable, except A2, A5 and A6. Peptides covering the central part of the protein such as A9 to A14 were predicted as stable in comparison to N and C terminal peptides. The aliphatic index was not skewed along the length of AdLP-D1. The aliphatic index indicated better stability over a range of temperatures and negative GRAVY values showed that overall, all AdLP-D1 peptides were hydrophilic and had strong interactions with water molecules. The antigenicity analysis of the peptides by VaxiJen^27^ revealed that these are all highly antigenic, with values above the threshold of 0.4 (Table 1). In the current study, we did not find any correlation between physiochemical properties and immunogenicity of the peptides.

**Table 1:** Summary of predicted physiochemical properties of peptides^24,25^.

Peptide	Sequence	MW*^26^	pI**	Negatively charged^aa^	Positively charged^aa^	Instability index^#^	Aliphatic index^##^	Grand average of hydrophobicity (GRAVY)^^^	Antigenicity (threshold 0.4)^^^^
A1	SSSDLSDSSISNDYLVANSG	2018	3.42	3	0	48.02	78	− 0.375	0.7241
A2	SNDYLVANSGDDSVASSSAS	1946	3.42	3	0	38.39	63.5	− 0.36	1.0194
A3	DDSVASSSASSSIAADDSDL	1900	3.24	5	0	63.04	73.5	− 0.21	1.0257
A4	SSIAADDSDLSNNASSSNVN	1968	3.42	3	0	43.78	68.5	− 0.61	0.999
A5	SNNASSSNVNFENEVLSTNN	2141	3.79	2	0	13.73	53.5	− 0.97	0.9475
A6	FENEVLSTNNNEDTESEIVK	2311	3.83	6	1	36.94	68	− 1.12	0.7911
A7	NEDTESEIVKDSKNQLSSSS	2197	4.18	5	2	79.2	53.5	− 1.44	0.739
A8	DSKNQLSSSSLQASTKTKTT	2112	9.7	1	3	60.55	44	− 1.195	1.1344
A9	LQASTKTKTTLKGSGSSVYR	2113	10.46	0	4	14.71	58.5	− 0.71	1.2187
A10	LKGSGSSVYRGNPYYVTLTD	2177	8.43	1	2	− 9.59	68	− 0.495	1.0281
A11	GNPYYVTLTDSNGKVLASQK	2155	8.43	1	2	9.00	73	− 0.60	0.7261
A12	SNGKVLASQKVTFNILGKNY	2182	10	0	3	28.16	92.5	− 0.25	0.4959
A13	VTFNILGKNYTRTTDSKGVA	2185	9.7	1	3	39.92	73	− 0.36	1.3222
A14	TRTTDSKGVASININLAKGK	2074	10.29	1	4	28.37	83	− 0.53	1.5786
A15	SININLAKGKYNIACLYAGT	2127	9.1	0	2	84.14	112.5	0.29	0.8793
A16	YNIACLYAGTENYASSKLSV	2167	5.99	1	1	97.52	88	0.12	0.7904
A17	ENYASSKLSVALTVNLMSTK	2156	8.59	1	2	48.69	97.5	0.055	0.8185

*MW (molecular weight in g/mol).

**pI (isoelectric point).

^#^Instability index (value of instability index smaller than 40 is predicted as stable, a value above 40 predicts that the peptide may be unstable).

^##^Aliphatic index (larger values represent increase of thermostability of the peptide).

^^^GRAVY (positive GRAVY values indicate hydrophobic nature of peptide and negative values indicate hydrophilic nature).

^^^^Antigenicity index (values represent peptide antigenicity).

### AdLP-D1 homology-based model

**Figure 4:**
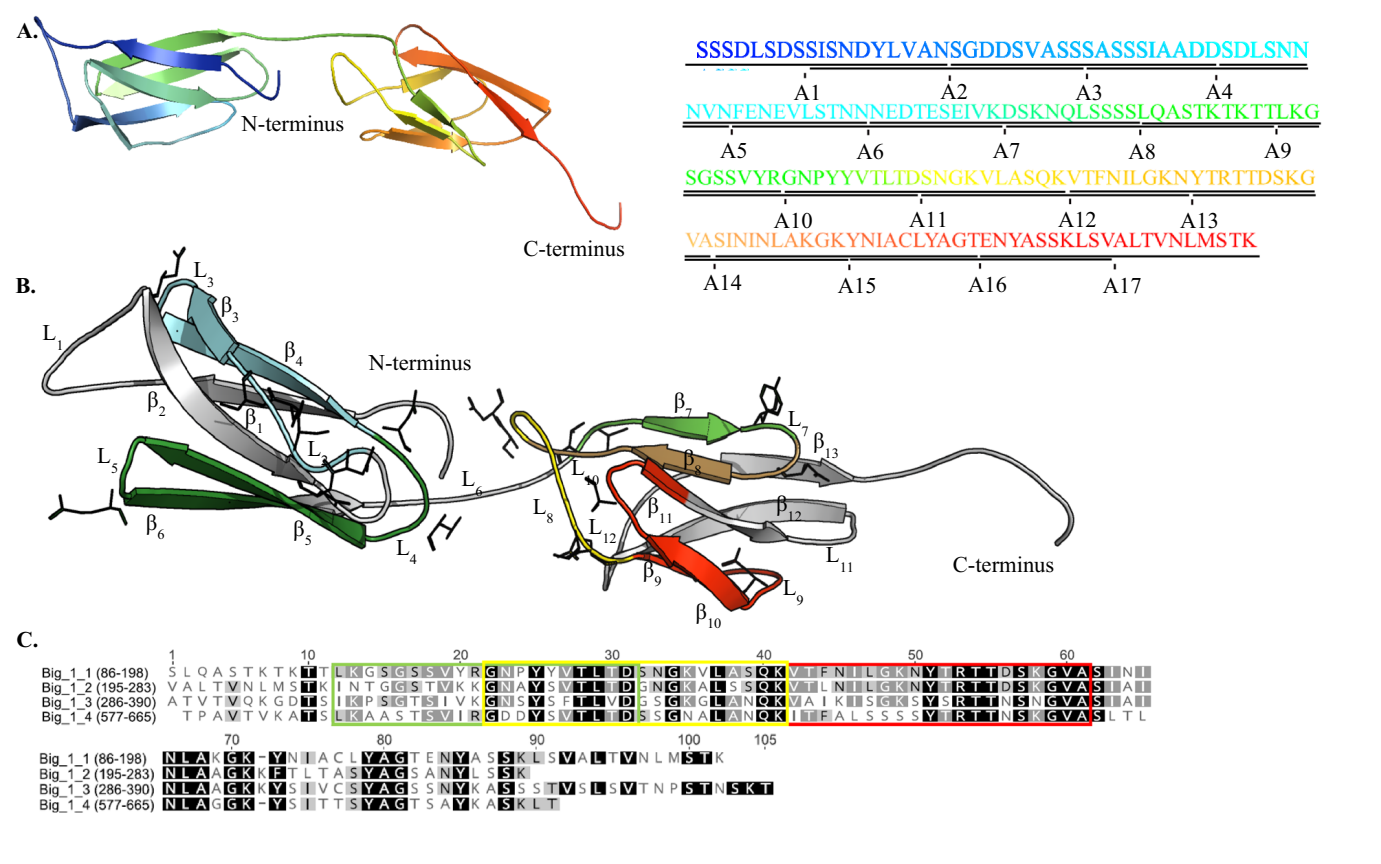
(**A**) The structure of AdLP_D1 was created using the online modelling server CPH version 3.2^28^ and a crystal structure of a bacterial integrin binding protein (PDB code 1CWV)^29^. The AdLP-D1 secondary structure and peptide sequences are highlighted with the identical colors. (**B**) Positions of the immunogenic peptides A5 (cyan), A7 (green), A10 (light green), A11 (yellow) and A13 (red) on the structure of AdLP-D1. The overlapping region between A10 and A11 is in light brown. Polar and charged residues are also shown as side chains. (**C**) Multiple amino acid sequence alignment of the Big_1 domains of Mru_1499 using MUSCLE^51^ alignment. Residues of the immunogenic peptides A10, A11 and A13 are highlighted in green, yellow and red boxes respectively.

To compare the structural features of the epitope-mapped region of AdLP-D1 and the availability of these epitopes for the antibodies to bind to the target protein, a highly reliable homology-based model of AdLP-D1 (Z-score of 10.3 and 97.2% sequence coverage) was retrieved using the CPH modelling server version 3.2^28^. This model was based on the crystal structure of a bacterial integrin binding protein (PDB code 1CWV^29^; Fig. 4A). The structure itself is made up of two domains comprised entirely of anti-parallel β-sheets, with six in the N-terminal domain (β_1_–β_6_, residues 6–83) and seven in the C-terminal domain (β_7_–β_13_, residues 93–179). These two domains are connected by a long loop (L_6_; residues 84–92), while the loops between the β-sheets remain relatively short excluding the large loops in either domain corresponding to residues 17–27 (L_1_) and 107–121 (L_8_) connecting β_1_–β_2_ and β_8_–β_9_ respectively. Peptides that generated stronger B- and T-cell responses are highlighted on the AdLP-D1 structure (Fig. 4B) and we observed that peptides such as A7 and A10 form tightly associating β-sheets within the macrostructure. Peptide A10 possesses a number of bulky solvent exposed side chain residues including Arg100, Tyr 99 and Tyr105 and an exceptional instability index value (− 9.59, Table 1).

Amino acid sequence alignment of the four Big_1 domain of Mru_1499 using the MUSCLE alignment tool^30^ revealed that the N-terminus Big_1 domain was most similar to other Big_1 domains of Mru_1499, with 58.7% amino acid identity to the second Big_1 at residues 195–283, 51.5% to the third Big_1 domain at residues 286–390, and 55.1% to the fourth Big_1 domain at residues 577–665 (Fig. 4C). Regions similar to overlapping peptides A10 and A11, which induced B- and T-cell responses in mice, were found in all 4 Big_1 domains (Fig. 4C) of AdLP.

### B-cell epitope prediction

**Figure 5:**
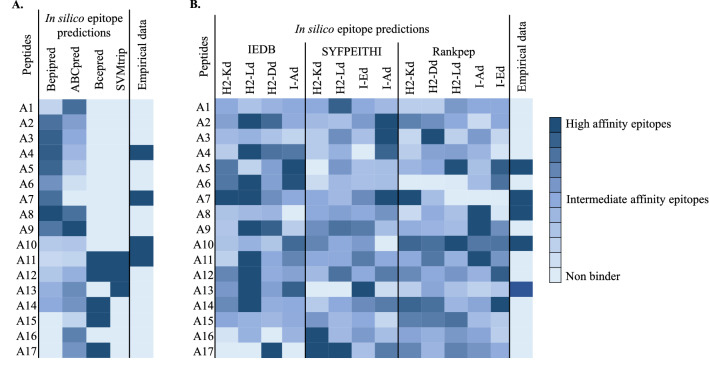
Heat maps depicting the predicted binding affinities of the AdLP-D1 B and T-cell epitopes for peptides A1–A17. (**A**) B-cell epitopes. Predictions were made using BcePred^31^ 1.0 server, ABCpred^18^, SVMtrip^20^ and BepiPred^32^. (**B**) T-cell epitopes. Predictions were made based on their binding affinities towards MHC class I and MHC class II alleles using IEDB^15^, SYFPEITHI^14^ and Rankpep^13^. The peptides empirically mapped as B-cell or T-cell epitopes are shown alongside the predictions.

We predicted linear B-cell epitopes using the BcePred^31^ 1.0 server, ABCpred^18^, SVMtrip^20^ and BepiPred^32^. The results are displayed in Fig. 5A as color intensity depicting the prediction of strong (royal blue), intermediate (sky blue), and low binding (light blue) affinities of B-cell epitopes. Screening of B-cell epitopes of AdLP-D1 by these prediction programmes revealed over- and under-prediction of B-cell epitopes along the whole length of the protein. Four peptides, A4, A7, A10 and A11 were mapped as B-cell epitopes of AdLP-D1. Of these peptides, A4 and A7 were predicted as B-cell epitopes by BepiPred and A11 was predicted by BcePred and SVMtrip. Peptide A10 was not predicted as a B-cell epitope by any of the four programmes we used in this study, yet it stimulated a strong IgG1 and IgG2a antibody response.

### T cell epitope prediction

Three algorithms, IEDB^15^, SYFPEITHI^14^ and Rankpep^13^, were used to predict T-cell epitopes in AdLP-D1. Peptides sequences were submitted to these servers for MHC class 1 alleles H2-Kd, H2-Dd and H2-Ld, and for MHC class II alleles H2-IED and H2-IAd binding predictions. Of the three, the IEDB Analysis Resource database using the NetMHCpan prediction method generated quantitative prediction of the affinity of the peptides to both MHC class I and II interaction that were partially in agreement to our experimental data of T-cell epitopes (Fig. 5B). Although predicted MHC I and II restricted epitopes by IEDB were evenly distributed throughout the protein sequence (Fig. 5B), three of the five peptides (A5, A7, A8, A10 and A13) mapped as T-cell epitopes were predicted as T-cell epitopes with strong affinity by two or more programmes. For instance, Rankpep^13^ predicted A5 for MHC class I allele H2-Ld and MHC class II allele I-Ed, while IEDB predicted A5 for MHC class 1 allele H2-Kd with intermediate affinity and MHC class II allele I-Ad with strong affinity. Peptide A7 was predicted for MHC class I alleles (H2-Kd, H2-Ld) by IEDB, while SYFPEITHI^14^ predicted binding affinity of A7 for MHC class II alleles I-Ad (Fig. 5B). Peptide A10 was consistently predicted by Rankpep as a high affinity T-cell epitope for both MHC class I and II alleles. IEDB predicted peptide A13 for MHC class 1 allele H2-Ld and by SYFPEITHI^14^ for MHC class II allele I-Ed with high affinity.

## Discussion

Epitope mapping has been used in recent years to identify key epitopes of antigen molecules and guide the design of peptide-based subunit vaccines to induce effective immune reponses^33^. It is understood that promising vaccine candidates should have both B- and T-cell epitopes. Combination of these epitopes would generate specific and balanced immune responses against the targeted antigen and will provide long lasting immunity. Furthermore, insights into the function of the antigen in the target organism can guide vaccine design strategies towards humoral immunity generating neutralizing antibodies, or towards cellular immunity by revealing the immune effectors for protection^34^. Prediction of immunodominant epitopes on protein antigens with a high degree of accuracy can improve vaccine design and speed up vaccine development.

Currently available computational methods for B- and T-cell epitopes prediction have limited application due to their lack of accuracy, with a large number of false positives and poor predictions^11^. In this study, we identified B- and T-cell epitopes of the AdLP-D1 domain (residues 19–198) of the putative adhesin protein Mru_1499 from *Methanobrveibacter ruminantium* M1. Mru_1499 was previously identified as an adhesin that binds to a broad range of rumen protozoa and bacteria^22,35^. A detailed structure of this protein is not available but the protein is predicted to have four bacterial immunoglobulin-like (Big_1) domains and one C-terminal transglutaminase-like domain (TG)^22–24^. Only the first Big_1 domain has been reported to be required for protozoa binding^22^, and this was part of the recombinant AdLP-D1 used in our study. We compared immunogenicity data obtained in a mouse model to the various prediction tools available for B- and T-cell epitopes prediction.

Mice were immunized with peptide-based vaccines to map the epitopes of AdLP-D1 that interact with B- and T-cells, and structural modelling was used to support and compare the results. B-cell epitopes identified by serum IgG1 and IgG2a through ELISA suggested that three regions of AdLP-D1 regions were antigenically effective/immunogenic, and it could be inferred that these B-cell epitopes are important for triggering humoral immune responses. These were peptide A4 (residues 49–68), peptide A7 (residues 79–98) and peptides A10 and A11 (residues 109–138).

Antibody secretion by B-cells is a direct consequence of cytokine release^36^, including IFNγ and IL-17A. IFNγ is a Th1 cytokine, involved in the modulation of various immunoglobulin classes^37^. Similarly, IL-17A is important for protective immunity against extracellular pathogens and also for the clearance of intracellular pathogens^38,39^. Knowledge of the dominant T-cell epitopes of an antigen is a crucial step to developing a T-cell-targeted vaccine. Helper CD4+ and CD8+ T-cells that release IFNγ and IL-17A activate immune cells^40,41^. Consequently, epitopes recognized by mouse IgGs and possibly presented via MHC II may be associated with B-cell predictions through crosstalk with APCs^42^. In the current study, we observed that mice vaccinated with peptide-based vaccines exhibited antigen-specific IFNγ and IL-17 responses (Fig. 3). The apparent higher background IFNγ responses observed with the recombinant protein may be due to the presence of *E. coli* LPS/endotoxin in the protein preparation. However, certain peptides resulted in large and statistically significant IFNγ responses while others did not, indicating this was not a significant factor in the interpretation of the data. Identification of T-cell epitopes of AdLP-D1 by cytokine profiling revealed that peptides A5, A7 A8, A10 and A13 induced significant cytokine responses upon re-stimulation of splenocytes with peptides and protein.

These immunogenic regions representing B- and T-cell epitopes of AdLP-D1 were checked for their localization in the homology-based model of the protein. Validated epitopes were found in the outside region of the protein with loop like secondary structure reflecting their flexibility and accessibility to the antibodies (Fig. 4B). The 3-dimensional structure of AdLP-D1 based on homology was modelled on the crystal structure of bacterial integrin-binding protein^29^. Interestingly, further analysis of the AdLP-D1 modelled structure revealed that immunogenic accessible peptides A5, A7, A10 and A11 are composed of β-strands and loops. Moreover, peptides A7 and A10 appear to form stable secondary structure in solution with two beta sheets and large exposed loop sections. These loop regions have large sections of solvent exposed residues including the larger side chains of tyrosine, arginine and asparagine. We suggest that the tightly associating β-strands in the predicted structure have increased structural stability for these peptides in solution and generate greater antibody responses as reported for *E. coli*-secreted protein B^43^. Additionally, peptides A10 and A11 were found well conserved in all the 4 Big_1 domains of Mru_1499, reflecting that Ig-like fold are evolutionary ancient protein folds found in proteins with diverse function^44,45^.

Linear B-cell epitopes are easier to predict by their amino acid sequences using bioinformatics tools, as no prior knowledge of protein conformational structure is required. A large number of linear B-cell epitopes can be predicted from complete genomes^46^. We combined multiple bioinformatics tools of epitope prediction, to characterize and allow selection of the best linear B-cell epitope candidates for comparison with experimentally mapped epitopes of AdLP-D1. Although four different in silico tools, based on distinct algorithms, were used to identify potential linear B-cell epitopes of AdLP-D1, the predicted epitopes did not fully match with the linear B-cell epitopes identified by peptide antibody interactions in the in vivo approach (Fig. 5A). For example, ABCpred predicted a set of epitopes represented by peptides A1, A8, A9, A13, A14, A16, and A17, but these were different from the epitopes identified experimentally, i.e., A4, A7, A10, and A11. Only two peptides recognized by mouse antibodies, A4 and A7, matched the predictions made by BepiPred. Interestingly, the whole N-terminal region of AdLP-D1 was predicted to be immunogenic by BepiPred, from peptides A2 to A9. In addition to the widely used ABCpred and BepiPred algorithms, we tested the BcePred and SVMtrip B cell epitope prediction methods as well. Disappointingly, the results of these two additional methods did not show noteworthy improvements over the previous methods. The only epitope predicted by both BcePred and SVMtrip that was confirmed by experimental data was peptide A11. In contrast to BepiPred, most of the peptides predicted by BcePred are part of C terminal end of the AdLP-D1. Thus, predictions made by the algorithms used in this study provided contradictory results and none of their results fully matched with the experimental findings.

The in silico methods used are based on different models and algorithms such as a combination of a Hidden Markov Model (HMM) with an amino acid propensity scale used in BepiPred, a Neural Networks model in ABCpred, Support Vector Machines (SVM) models in SVMtrip and BcePred. Each differs from the other on feature selection, data set curation and SVM specific parameters^47^. Each of these in silico methods, while trained on data sets covering a wide range of virus, bacteria, protozoa and fungi proteins, will have limits on their ability to correctly predict B-cell antigenic epitopes in methanogen proteins with few 3-dimensional structures available^48^. This suggests that further training and validation of these prediction methods on methanogen proteins is needed to predict B-cell epitopes.

Computational approaches for prediction of T-cell epitopes have been shown in the past to have potential for epitope discovery and vaccine design. Available databases for epitope prediction already cover a very wide range of peptides presented by MHC molecules. In this study T-cell epitopes predicted by IEDB, SYFPEITHI and Rankpep revealed several CD4+ (MHC class II) and CD8+ (MHC class I) epitopes distributed over the entire AdLP-D1 molecule. In contrast to in vivo cytokine production analysis, which indicated only few immunogenic epitopes represented by peptides A5, A7, A8, A10 and A13 in mice, similar to an earlier study which reported that of peptides predicted to bind to MHC molecules, only ~ 10% were shown to be immunogenic through in vivo studies ^11^. Using the current over predictions of these in silico tools, it is difficult to construct an accurate T-cell epitope map of AdLP-D1. Thus, T-cell epitope prediction algorithms are limited by their large number of false positives and false negatives as we observed in this study, where predicted potential epitopes failed to elicit experimental immune response and vice versa. A consensus method needs to be used to overcome the problem of limited capability of the individual predictive tools^49^. For methanogen proteins, MHC class I and II alleles peptide binding prediction algorithms could succeed if these programmes were trained on the experimental data of epitope discovery of archaeal proteins. An integrated approach, combining in vitro and in vivo methods could be the optimal pipeline for epitope mapping of methanogen proteins.

## Conclusion

In this study, B- and T cell-epitopes of AdLP-D1 were determined using both in vivo and in silico techniques. The majority of linear B-cell and T-cell epitopes identified empirically mapped to regions of an AdLP-D1 model protein showing high accessibility for inducing an immune response. Current in silico computational methods used for predicting B -cell and T-cell epitopes had varying degrees of accuracy for predicting linear B- and T-cell epitopes in AdLP-D1. It may be possible to improve the accuracy of these predictions by training the algorithms using data from in vivo studies performed on additional methanogen proteins.

## Materials and methods

### Recombinant protein production and purification

The DNA sequence corresponding to amino acids 19–198 of Mru_1499 from *Methanobrevibacter ruminantium* M1^22^ (GenBank accession no. WP_048812472.1, referred to here as AdLP-D1) with the addition of codons for a C-terminal hexahistidine (His) tag were synthesized and cloned into the pET-30a vector by GenScript (Piscataway, NJ, USA). The plasmid was transformed into *E. coli* BL21 (DE3) cells and the transformants were selected on plates of terrific broth^50^ supplemented with 50 µg/mL kanamycin (Sigma Aldrich, Auckland, New Zealand). Recombinant protein production was induced by adding 1 mM isopropyl β-d-1-thiogalactopyranoside to cultures that had been grown in 2 L liquid terrific broth medium at 37 °C until the OD_600_ reached ~ 1.2, and then incubating for a further 16 h at 15 °C. The cells were then pelleted by centrifugation at 10,000 × *g* for 10 min at 4 °C. Cells were re-suspended in PBS buffer (NaCl, 137 mM; KCl, 2.7 mM; Na_2_HPO_4_, 10 mM; KH_2_PO_4_, 1.8 mM, pH 7.4 with HCl) and disrupted using a microfluidizer (Microfluidics M-110P; Westwood, MA, USA). Insoluble cell debris was removed by centrifugation at 15,000 × *g* for 20 min at 4 °C. His-tagged protein was purified by affinity chromatography using precharged Ni sepharose columns following the manufacturer’s instructions (GE HealthCare, Danderyd, Sweden). Eluted fractions were analyzed by SDS-PAGE followed by Western blotting using anti-His tag antibodies (ThermoFisher Scientific, Auckland, New Zealand) and mass spectrometry (MS) using matrix-assisted laser desorption ionization–time of flight mass spectrometry (MALDI-TOF MS) for protein conformation. Mass spectrometry data was analyzed by Mascot software^51^. Eluted fractions containing AdLP-D1 protein were pooled and dialyzed against PBS and stored at − 80 °C until further use.

### Peptide design and synthesis

A series of 17 overlapping 20-mer peptides with an overlap of 10 aa residues between two adjacent peptides were designed to cover AdLP-D1. Two sets of peptides were synthesized by GenScript (Piscataway, NJ, USA) with > 95% purity. C-terminal Keyhole Limpet Haemocyanin (KLH) modified peptides via a cysteine residue (additional cysteine residue was added to the C-terminal end of each peptide for KLH conjugation) were used for vaccination, and N-terminal biotinylated peptides were used in ELISA. Peptides were dissolved in pure water just prior to use based on recommendations of their solubility provided by GenScript.

### Use of experimental animals

All procedures involving animals were performed in compliance with the Animal Welfare Act regulations of New Zealand, particularly the Animal Welfare Act 1999 (the Act) and the Animal Welfare (Records and Statistics) Regulations 1999, and were approved prior to the study by the AgResearch Grasslands Animal Ethics Committee, Palmerston North, New Zealand (approval number 14408).The authors have complied with ARRIVE guidelines^52^.

### Immunization of mice

Six-to-eight week old female BALB/c mice (n = 76) were supplied by the AgResearch Small Animal Facility (Hamilton, New Zealand), and kept in a biosecurity containment-2 room (under 21 °C and 50% humidity) at the Ulyatt Reid Small Animal Facility, AgResearch (Palmerston North, New Zealand) during the study. Mice were housed in groups of 4 per cage in plastic cages on a 12-h light/dark cycles and fed mouse pellets (Prolab^®^ RMH 1800; LabDiet, Richmond, IN, USA) and tap water (autoclave under pressure at 121 °C for 15 min) ad libitum. All animal experiments were approved by the AgResearch Grasslands Animal Ethics Committee, Palmerston North, New Zealand (approval number 14408). At the time of vaccination mice had body weights of 25.1 ± 0.2 g, (mean ± SEM).

Seventeen groups (n = 4 each) of BALB/c mice were vaccinated subcutaneously at the back of the neck with a 200 µL dose of a single peptide vaccine, consisting of 40 µg peptide mixed with Montanide ISA61 adjuvant (SEPPIC, Paris, France). One further group (n = 4) was vaccinated with recombinant AdLP-D1 (expressed in *E. coli*) and a control group (n = 4) was given adjuvant alone. Animals were vaccinated 3 times by the subcutaneous route at 2-week intervals.

### Cell preparation and immunological responses

Mice were euthanized two weeks after the last vaccination by using 100% CO_2_ inhalation at 30% flow rate for 20 min (according to AVMA Guidelines for the Euthanasia of Animals^53^) and cervical dislocation. Euthanized mice were dissected aseptically, and their spleens were removed to prepare a single cell suspension as described elsewhere^54^. Briefly, spleens of each animal were collected in 1 mL of Rosewell Park Memorial Institute Medium (RPMI^55^; Sigma-Aldrich, Auckland, New Zealand), supplemented with l-glutamine (0.3 g/L) and sodium bicarbonate, and kept on ice until used. Single cell suspension of splenocytes was prepared by gently pushing the spleen through a cell strainer with a mesh size of 100 µm (Corning, Glendale, CA, USA) using the 5 mL syringe plunger. Tissue was further disintegrated by aspirating it through a 23-gauge hypodermic needle 5 times. Red blood cells were lysed using a solution of 17 mM Tris–HCl and 140 mM NH_4_Cl, pH 7.2. After washing, cells were resuspended in RPMI supplemented with 10% (v/v) heat inactivated fetal bovine serum (Gibco Life Technologies, Auckland, New Zealand) containing 100 U/mL penicillin (ThermoFisher Scientific, Auckland, New Zealand), 100 µg/mL streptomycin (ThermoFisher Scientific, Auckland, New Zealand) in triplicate wells of flat-bottomed 96-well plates at a concentration of 5 × 10^5^ cells/well in a 200 μL volume. The cells were stimulated with medium alone as a negative control or in medium containing their respective vaccinated peptide/nonspecific peptide (at 5 μg/mL) or recombinant protein AdLP-D1/nonspecific protein (at 10 μg/mL), or staphylococcal enterotoxin B (100 ng/mL; Sigma-Aldrich) as a positive control. Plates were incubated at 37 °C in an atmosphere of 5% CO_2_ in air for 3 days. Following incubation, culture supernatants were carefully recovered by centrifugation at 200 × *g* for 10 min and stored at − 20 °C until analyzed for cytokine release by ELISA. Levels of antigen-specific IFNγ and IL-17A were measured in culture supernatants using sandwich ELISA kits (BD Biosciences, San Diego, CA, USA and BioLegend, San Diego, CA, USA, respectively), according to the manufacturers’ instructions.

### Measurement of antigen-specific antibodies in serum

Sera were collected from mice 2 weeks after final immunization to measure peptide and recombinant protein-specific immunoglobulin (IgG1 and IgG2a) responses by ELISA. Briefly, 100 ng/well recombinant protein was used for coating Microlon high-binding plates (Greiner Bio-One, Frickenhausen, Germany) and biotinylated peptides (50 ng/well) were used to coat streptavidin-coated high-capacity plates (Pierce Biotechnology, Rockford, IL, USA). Serum from individual mice was diluted, added to the plates, and incubated for 2 h at room temperature. After incubation, the plates were washed four times with washing buffer (0.5% [vol/vol] Tween-20 in PBS) with 3 min soaking during each wash. Diluted goat anti-mouse IgG1 or IgG2a antibodies (1:6000) conjugated with horseradish peroxidase (ICLLab, Portland, OR, USA) were added to the plates and incubated for 1 h at room temperature. The plates were washed and 50 µL of 3,3′,5,5′-tetramethylbenzidine substrate (BD Biosciences, San Diego, CA, USA) was added to each well and incubated for 30 min at room temperature. The reaction was stopped by adding 0.5 M H_2_SO_4_ to each well and the OD at 450 nm was measured using a microplate reader (VERSAmax; Molecular Devices, Sunnyvale, CA, USA) to detect the IgG isotypes. Data were presented after subtraction of the background OD as antibody titer measured for each mouse along with their geometric mean in response to peptides and recombinant protein.

### In silico analysis of the physicochemical properties of peptides

Physiochemical properties of peptides like molecular weight, theoretical isoelectric point (pI), and amino acid composition were analyzed using the Expasy ProtParam tool^26^ (http://web.expasy.org/protparam/). Antigenicity of each peptide was determined using VaxiJen 2.0 server^27^ (http://www.jenner.ac.uk/VaxiJen), with the threshold for predicting the probable antigen set at 0.4.

### Homology modelling and validation

The online homology modelling tool CPHmodels version 3.2^28^ was used for modelling the structure of the AdLP-D1. MUSCLE^30^ web server was used for multiple alignment of antigenic peptides of AdLP-D1 with the rest of three Big-1 domains of Mru_1499. Figures were prepared with PyMol^56^ and Geneious Pro^57^ sequence analysis software.

### In silico linear B-cell epitope prediction

The AdLP-D1 protein sequence was screened for linear B-cell epitopes using the Immune Epitope Database (IEDB) Analysis Resource tools (http://tools.iedb.org/bcell/) including BepiPred 1.0 server^32^. The threshold for predicting the probable antigen was set at default threshold of 0.5. The complete protein sequence was further screened for the prediction and analysis of linear B-cell epitopes using ABCpred^18^, BcePred 1.0 server^31^, and SVMTrip^20^. The window size was set at 20 and the proportion of residues at regions corresponding to the epitopes above the default thresholds calculated.

### In silico T-cell epitope prediction

The BALB/c mice MHC class 1 and II alleles of T-cell epitopes were predicted using the IEDB Analysis Resource tool (http://www.iedb.org/mhc)^15^, SYFPEITHI^14^ and Rankpep^13^. The binding affinity of peptides (20-mers) to MHC class 1 alleles H2-Kd, H2-Dd and H2-Ld, and MHC class II alleles H2-IED and H2-IAd of BALB/c mice were predicted.

### Statistical analysis

The significance of differences between two groups was analyzed using One-Way ANOVA followed by a Dunnett’s posthoc multiple comparison test^58^. All data are expressed as the mean ± SE and plotted using Minitab V.18.1 (Minitab Inc., USA). The level of significance was set at *P*-value of ≤ 0.05.

## Supplementary Information

Supplementary Figures.
