# Supplementary material for: Mapping immunogenic epitopes of an adhesin-like protein from Methanobrevibacter ruminantium M1 and comparison of empirical data with in silico prediction methods
Source: Sci Rep. 2022 Jun 21;12:10394. doi: 10.1038/s41598-022-14545-8 (PMC9213418; doi:10.1038/s41598-022-14545-8)
Supplement: Supplementary file 1 — Supplementary Figures. [file 41598_2022_14545_MOESM1_ESM.pdf]

Mapping immunogenic epitopes of an adhesin-like protein from *Methanobrevibacter ruminantium* M1 and comparison of empirical data with *in silico* prediction methods

Sofia Khanum[1*], Vincenzo Carbone[1], Sandeep K. Gupta[1], Juliana Yeung[1], Dairu Shu[1], Tania Wilson[1], Natalie A. Parlane[1], Eric Altermann[1,2], Silvia M. Estein[3], Peter H. Janssen[1], D. Neil Wedlock[1] and Axel Heiser[1]

[1]AgResearch, Palmerston North, New Zealand

[2]Riddet Institute, Massey University, Palmerston North, New Zealand

[3]Centro de Investigación Veterinaria de Tandil (CIVETAN), UNCPBA-CONICET-CICPBA, Facultad de Ciencias Veterinarias, Campus Universitario, (7000) Tandil, Argentina.

*Corresponding author: Sofia Khanum, Scientist, AgResearch, Palmerston North, New Zealand. sofia.khanum@agresearch.co.nz.

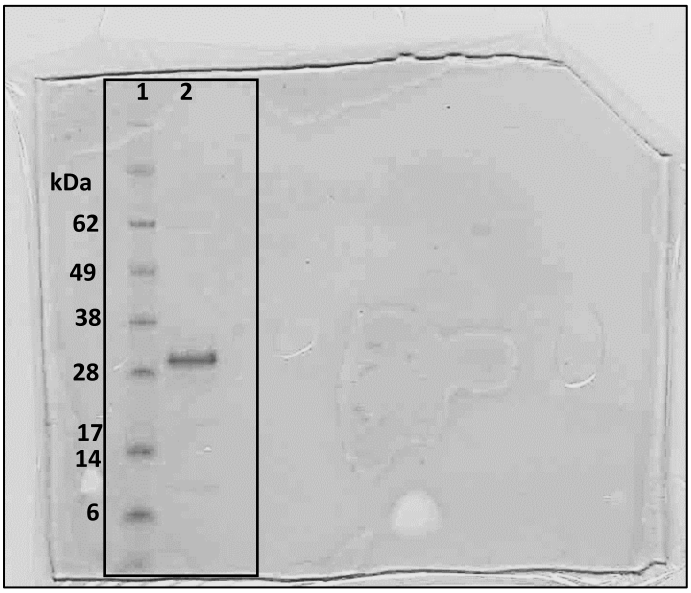

Supplementary Figure 1. 1 µg of purified AdLP-D1 was mixed with 4 × SDS sample buffer and heated for 5 min at 95°C followed by loading on the 10% SDS gel (Bolt^{TM} 10% Bis-Tris Plus Gels; Invitrogen, Carlsbad, CA, USA) and run for 40 min at 150 V. After electrophoresis, separated proteins were stained with SimplyBlue SafeStain (Thermo Fisher Scientific, Auckland, New Zealand) for 1 h followed by de-staining four times for 30 min each with water. Imaging of the Coomassie-stained gel was performed on a ChemiDoc imaging system (Bio-Rad, Auckland, New Zealand) fitted with a white light conversion screen. The image was processed using Image Lab Touch Software (Bio-Rad). Bands were detected with a sensitivity setting of 50 for Coomassie-stained gels. The region of interest (shown as a black rectangle) was cropped. Lane1, SeeBlue Plus2 Pre-Stained Protein Standard (Thermo Fisher Scientific); Lane 2, purified AdLP-D1. The numbers to the left of the protein markers are protein molecular sizes in kDa.

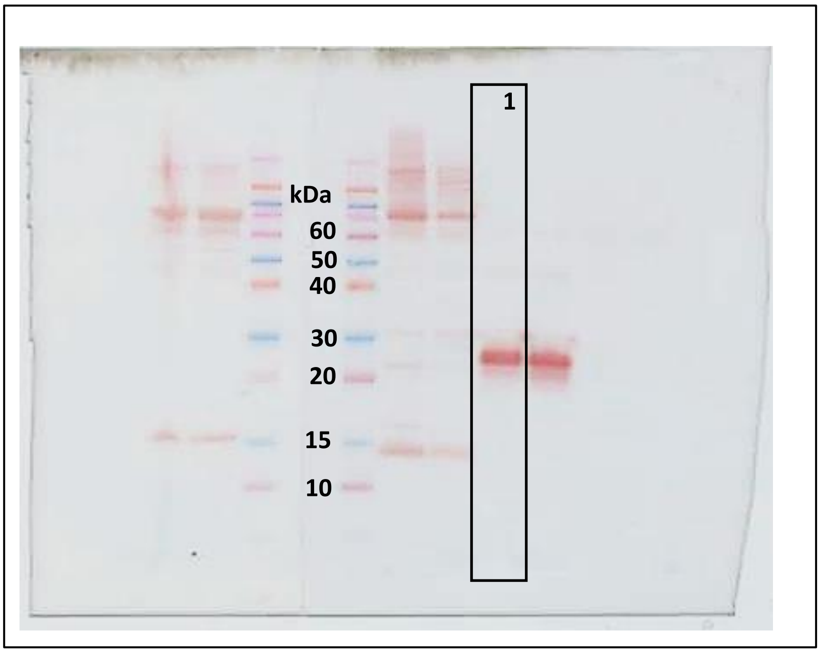

Supplementary Figure 2. The AdLP-D1 recombinant purified protein gel was transferred onto a PVDF membrane and incubated for 2 h at room temperature in blocking solution containing 5% (w/v) skim milk, 50 mM Tris-Cl, 150 mM NaCl and 1% (w/v) Tween 20 (TBST-pH 7.4) for blocking. Hexahistadine-tagged AdLP-D1 protein was detected using monoclonal primary antibodies against the hexahistidine tag (Thermo Fisher Scientific) at 1:1000 dilution. The membrane was washed three times with TBST and incubated with secondary HRP conjugated anti-mouse antibodies (Dako, Kyoto, Japan) at 1:1000 dilution for 1 h at room temperature. The membrane was then washed three times with TBST and protein was visualized using 0.05% (w/v) 3-amino-9-ethylcarbazole and 0.015% (v/v) H_2O_2 in 50 mM acetate buffer, pH 5.5). The region of interest (shown as a black rectangle) was cropped. Protein marker; Novex Sharp Pre-Stained Protein Standard (Invitrogen, Carlsbad, CA, USA); Lane 1, purified AdLP-D1. The numbers to the left of the protein markers are protein molecular sizes in kDa.

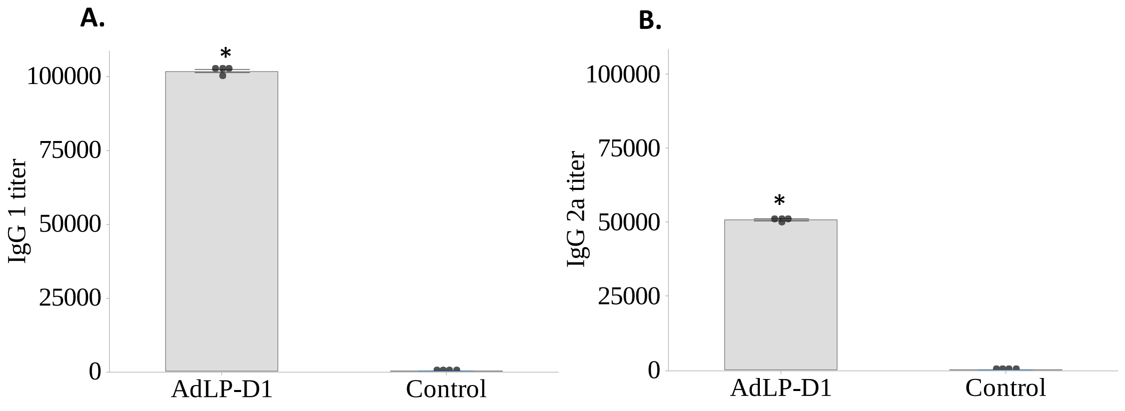

Supplementary Figure 3. IgG1/IgG2a titers in mouse sera against the recombinant AdLP-D1 protein. Individual data points are represented as black dots, while the bars represent the geometric means (± SE) of sera from four mice. **A**, IgG1titers in sera from mice vaccinated with the recombinant protein AdLP-D1 or in sera from control mice. **B**, IgG2a titers against the recombinant protein AdLP-D1 in sera from mice vaccinated with the recombinant protein AdLP-D1 or in sera from control mice. Significance differences to the control group are shown as * $P < 0.05$.
